# Resource Allocation and Data Offloading Strategy for Edge-Computing-Assisted Intelligent Telemedicine System

**DOI:** 10.3390/s23104943

**Published:** 2023-05-21

**Authors:** Yan Li, Yubo Wang, Shiyong Chen, Xinyu Huang, Tiancong Huang

**Affiliations:** 1 School of Microelectronics and Communication Engineering, Chongqing University, Chongqing 400044, China; 202112131133@stu.cqu.edu.cn (Y.L.);; 2 Beijing Smart-Chip Microelectronics Technology Co., Ltd., Beijing 100192, China

**Keywords:** intelligent telemedicine, edge computing, resource allocation, data offloading

## Abstract

Intelligent telemedicine technology has been widely applied due to the quick development of the Internet of Things (IoT). The edge-computing scheme can be regarded as a feasible solution to reduce energy consumption and enhance the computing capabilities for the Wireless Body Area Network (WBAN). For an edge-computing-assisted intelligent telemedicine system, a two-layer network architecture composed of WBAN and Edge-Computing Network (ECN) was considered in this paper. Moreover, the age of information (AoI) was adopted to describe the time cost for the TDMA transmission mechanism in WBAN. According to the theoretical analysis, the strategy for resource allocation and data offloading in edge-computing-assisted intelligent telemedicine systems can be expressed as a system utility function optimizing problem. To maximize the system utility, an incentive mechanism based on contract theory (CT) was considered to motivate edge servers (ESs) to participate in system cooperation. To minimize the system cost, a cooperative game was developed to address the slot allocation in WBAN, while a bilateral matching game was utilized to optimize the data offloading problem in ECN. Simulation results have verified the effectiveness of the strategy proposed in terms of the system utility.

## 1. Introduction

The rapid popularization of IoT technology has accelerated the application over a range of industries, allowing for the change from classical informatization to automation and intelligence. Intelligent telemedicine as an IoT technology applied in the medical field is dedicated to raising the level of medical care [1]. Conventional medical services struggle to meet the demands of many patients due to resource limitations, complicated procedures, and high expenses. For the elderly and chronic patients, inconvenient medical consultations, health monitoring, and medical care result in opportunities for effective treatment and disposal being missed [2]. It can enhance the transmission and processing capabilities if IoT technology is applied in traditional medical services. Moreover, the real-time monitoring of a patient’s health state can be achieved by deploying a variety of heterogeneous medical sensors on patients.

Telemedicine technology can offer universal health monitoring services. However, the increase in the number of patients can overrun the operation system of the medical center (MC). Additionally, the delay-sensitive requirements of medical information analysis cannot usually be met by local gateway user nodes (GUNs) with poor computing capability, which restricts the advancement of the intelligent telemedicine system. As an emerging distributed computing framework, edge computing can help decrease the local latency of processing medical data packets and enhance energy efficiency by offloading data from the GUNs to ESs and avoiding medical data transmission to the MC, which usually leads to a larger latency for data processing [3]. Therefore, the edge computing utilized in intelligent telemedicine systems can effectively reduce the energy consumption of local medical data analysis or the transmission delay from the GUNs to the MC. Furthermore, it can meet the real-time analysis demand of emergency medical monitoring data and improve the quality of service (QoS) for the intelligent telemedicine system.

In this paper, a resource allocation and data offloading strategy for edge-computing-assisted intelligent telemedicine systems was considered. To maximize the system utility, a contract mechanism was introduced to incentivize ESs to participate in system cooperation. Moreover, the whole framework for medical monitoring data updates was separated into two-layer networks: the WBAN and the ECN. For the WBAN, heterogeneous sensors share the channel through TDMA technology, and the GUNs minimize the AoI of each monitoring packet by slot allocation. For the ECN, the GUNs can choose the proper ESs for access based on the corresponding load and network conditions.

The main contributions of this article are summarized as follows:

- An edge-computing-assisted intelligent telemedicine system was studied. Considering the requirements of the patients and the utility of the MC, the system utility was determined by the utility of the MC and the system cost;
- To increase the contributions of ESs to the system, an incentive mechanism based on the contract theory was introduced to motivate ESs to provide communication resources;
- A two-layer communication network, encompassing the WBAN and the ECN, was used to describe the edge-computing-assisted intelligent telemedicine system. For the WBAN, a cooperative game was proposed to optimize the allocation of time slots. For the ECN, a bilateral matching game was introduced to address the data offloading issue.

The remaining parts of this paper are as follows. The second section introduces recent studies related to intelligent telemedicine technology. The third section introduces the system model studied in this paper. The fourth section introduces the optimization strategy to maximize system utility. In the fifth section, the performance of the strategy proposed is verified by simulation experiments. Finally, the sixth section summarizes the work of this paper.

## 2. Related Studies

As an important component of smart wearable technology and Internet of Medical Things (IoMT), WBAN technology is considered an important way to achieve intelligent telemedicine. The WBAN is a special wireless sensor network (WSN) that surrounds the human body and consists of GUN and micro-inertial sensors distributed on the surface or inserted into the human body [4]. Numerous researchers have offered various low-power short-range communication solutions based on the IEEE 802.15.6 standard [5]. Different channel access strategies based on TDMA technology have been introduced in [6,7,8,9] to meet the requirements of the patients, which can improve energy efficiency and enhance the utility of the WBAN system. A hybrid periodic protocol was designed to achieve efficient resource allocation and emergency control in adaptive continuous non-competitive periods in [6]. In [7], to improve the reliability of emergency data, an adaptive channel access scheme based on a super-frame structure was designed. The energy consumption of successfully transmitted data frames under this scheme was examined, and an analytical model for calculating the average delay and reliability of emergency data frames was suggested. Two technologies were introduced to improve the reliability and energy efficiency of the WBAN in [8]. The first technology allowed nodes to adaptively allocate sleep time during active periods based on channel state to avoid deep channel fading. The second technology was dynamic slot allocation based on node requirements. In [9], two new TDMA-based strategies were proposed to improve the reliability and energy efficiency of the WBAN. All of them could adaptively synchronize the nodes when processing the channel state of the nodes. Moreover, in [10], a channel periodicity-based scheduling (CPBS) strategy was proposed to realize energy-efficient and reliable communication in dynamic WBANs. To improve the overall super-frame utilization by assigning dynamic slots, a dynamic slot allocation (DSA) scheme based on non-overlapping backoffs (NOBA) was utilized, which can reduce energy consumption [11]. In [12], a MAC protocol based on dynamic super-frame (SF) structure and priority-based dedicated slot allocation was proposed to enhance QoS and energy consumption.

The above studies can significantly improve the energy efficiency of the system with sufficient slots. However, when the slots are insufficient to meet the requirements of all sensors, the urgency of the packets should be seriously evaluated, otherwise resulting in the poor overall performance of the system. Moreover, with the widespread application of WBAN technology in medical health monitoring scenarios, many disadvantages have emerged, such as insufficient computing resources, high energy consumption, and short operational life. Edge computing is regarded as a feasible solution to solve these problems. In [13], latency and energy consumption were successfully decreased by transferring intensive computing tasks to the ESs in order to expand the computing capability of the WBNA system. To process large amounts of physiological data, an improved computation architecture by combining the WBAN system with edge-computing technology was suggested to obtain a shorter average service time and higher success rates for task execution in [14]. In [15], an alternating direction method of the multipliers-based algorithm was proposed to maximize the total payoffs of the ESs and the patients. In [16], a two-stage potential game-based computation offloading strategy for WBANs assisted by edge computing with consideration for the task and user priorities was proposed, which can meet the requirements of low delay and low energy consumption in the WBANs scenario. For task offloading and migration in WBAN systems, a collaborative optimization strategy based on the asynchronous advantage actor-critic (A3C) algorithm was suggested to dramatically enhance the QoS for the patients in [17]. Furthermore, to fully utilize the potential of edge computing, reasonable resource allocation mechanisms should be taken into account. In [18], two auction mechanisms for the blockchain network formed by edge computing services and miners were proposed to maximize social welfare. In [19], a stacked task sorting and ranking mechanism was proposed to improve resource allocation in each edge device.

Most of the reported studies have assumed that the system cost is composed of the data delay (generally including transmission delay and computation delay) and the energy consumption for transferring and computing the medical monitoring data, and the system utility is defined as the opposite of the system cost. In this paper, the utility of the MC is considered in the system utility for the intelligent telemedicine system. Moreover, as the generated packets usually experience a waiting delay before scheduled transmission in TDMA, the AoI is also considered in the system cost, which is different from the reported works. Additionally, a two-layer network architecture is proposed to describe the process of medical data updates. An incentive mechanism based on contract theory is considered to motivate the ESs to provide communication and computing resources to maximize the system utility. Furthermore, a cooperative game is proposed to allocate the time slots in the first layer network (the WBAN), and a bilateral matching game is adopted to solve the data offloading problem in the second layer network (the ECN).

## 3. System Model

**Figure 1:**
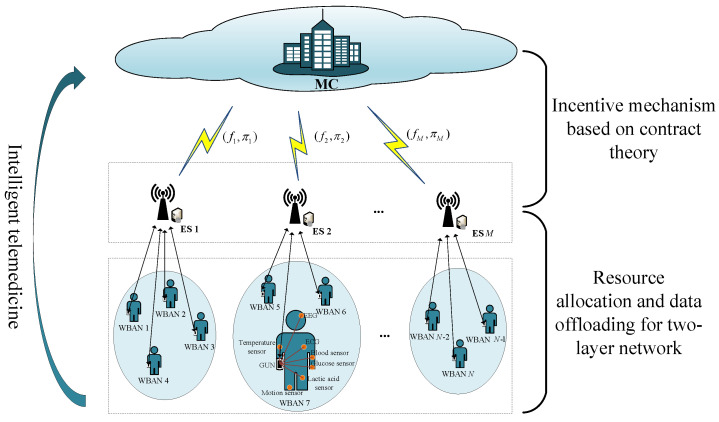
Edge-computing-assisted intelligent telemedicine system model.

In this paper, we considered an intelligent telemedicine scenario assisted by edge computing. As depicted in Figure 1, the computation offloading framework in the intelligent telemedicine system consists of four parts, i.e., medical sensors, GUNs, ESs, and the MC. To relieve the high energy consumption of GUNs, the contract mechanism for incentivizing ESs to participate in the cooperation was introduced in the MC. Thus, the whole system is described as a two-layer network architecture: the WBAN and the ECN. A local GUN and numerous sensors around the body make up a patient-centered WBAN. Each sensor in the WBAN keeps records of medical monitoring data and sends it to the associated GUN. The GUNs and the corresponding ESs make up the ECN. After gathering monitoring packets from the WBAN, the GUNs select the appropriate ES to transmit and analyze the medical monitoring data.

The assumption is that there are N patients and the number of patients is equal to the number of WBANs or GUNs, denoted by N={1,2,…,N}. Each WBAN consists of a GUN and S heterogeneous medical sensors deployed on the patient, denoted by S={1,2,…,S}. The corresponding medical analytic functions are carried out on the ESs, denoted by M={1,2,…,M}. Some parameters involved in this paper are listed in Table 1.

### 3.1. Interaction between MC and ESs

Considering that each ES has a different willingness to join the intelligent telemedicine system to provide the communication resources for GUNs’ access, the MC provides the contract composed of communication resources and rewards corresponding to the willingness of ESs. ESs with different participation willingness provide different communication resources and obtain corresponding rewards from the MC. θ is introduced to denote the participation willingness of different ESs. Thus, we can classify the ESs based on their participation willingness. If there are I types of participation willingness for the ESs, which are denoted as [20]:

(1) $$\theta_{1}<\theta_{2}<\ldots <\theta_{I}$$

where a larger value of θ indicates a stronger desire for the ES to assist the intelligent telemedicine system. It is assumed that the probability of the ES with participation willingness $\theta_{i}$ is $\xi_{i}$, and $\sum_{i=1}^{I}\xi_{i}=1$. In this paper, ES i belongs to type $\theta_{i}$ and type $\theta_{i}$ ES is ES i, i.e., we assume that the types’ number equals the ESs’ number. The contract provided by the MC for the ESs with participation willingness $\theta_{i}$ can be described as $\left(q_{i}(\theta_{i}),\pi_{i}(\theta_{i})\right)$, where $q_{i}(\theta_{i})$ denotes the communication resources provided by the ESs with participation willingness $\theta_{i}$, and $\pi_{i}(\theta_{i})$ is the corresponding rewards. It is free for the ESs to accept or decline the contract. If the ESs decline the contract, the contract will be expressed as $\left(q_{i}(\theta_{i}),\pi_{i}(\theta_{i}))=(0,0\right)$.

**The utility of the MC**: The ESs usually afford communication resources according to their participation willingness for GUNs’ access, which can be regarded as an auxiliary analysis to process the medical monitoring data. In order to enhance the contributions of the ESs, the MC can return the corresponding rewards to the ESs if they provide the appropriate communication resources. When the communication resource provided by the ESs for GUNs is $q_{i}(\theta_{i})$, while the corresponding reward provided by the MC is $\pi_{i}(\theta_{i})$, the utility function of the MC can be denoted as:

(2) $$U_{\mathrm{MC}}(i)=\alpha q_{i}(\theta_{i})-\pi_{i}(\theta_{i})$$

where *α* is the unit revenue obtained by the MC from the communication resources provided by ESs. Since there are I types of ESs with probability $\xi_{i}$, the expected utility of the MC is:

(3) $$U_{\mathrm{MC}}=\sum_{i=1}^{I}\xi_{i}[\alpha q_{i}(\theta_{i})-\pi_{i}(\theta_{i})]$$

**The utility of the ES**: The rewards obtained from the MC can compensate for the cost of the ESs providing communication resources. Thus, the utility function of the ES with participation willingness $\theta_{i}$ can be expressed as [21,22]:

(4) $$U_{\mathrm{ES}}(i)=\theta_{i}v(\pi_{i}(\theta_{i}))-cq_{i}(\theta_{i})$$

where *v(π)* is the evaluation function corresponding to the reward π, and the coefficient c represents the cost of providing unit communication resources. Without loss of generality, the evaluation function can be defined as v(π)=εln(1+π), and ε>0 is a constant coefficient [21].

### 3.2. Communication Model for the WBAN

Since medical information monitored by body sensors is time sensitive, AoI is used to describe the update speed of medical packets [23]. In this paper, TDMA technology was utilized to share the channel within the WBAN. Thus, the medical sensors can transmit the collected medical monitoring data according to the assigned time slots. To facilitate calculation, all data packets are generated with a timestamp $G_{ij}(t)$, where i∈N and j∈S. The AoI of each sensor is represented as the difference between the current time slot t and the timestamp $G_{ij}(t)$ when the latest medical monitoring data packet was updated. Let $A_{ij}(t)$ represents the AoI of the sensor j in WBAN i at slot t, and the AoI update process can be expressed as [24]:

(5) $$A_{ij}(t)=\{\begin{array}{l} A_{ij}(t)+1,\;\mathrm{otherwise}\\ t-G_{ij}(t),\;\mathrm{the}\;\mathrm{timestamp}\;\mathrm{is}\;\mathrm{updated} \end{array}$$

The uplink data transmission rate for the sensor j in the WBAN i can be expressed as:

(6) $$r_{ij}^{\mathrm{SN}}=w\log_{2}(1+\frac{p_{ij}^{\mathrm{SN}}h_{ij}^{\mathrm{SN}}}{\sigma^{2}})$$

where w represents the bandwidth of the channel for the WBAN i, $p_{ij}^{\mathrm{SN}}$ is the transmission power of the sensor j in WBAN i, and $h_{ij}^{\mathrm{SN}}$ is the channel gain between the sensor j and the corresponding GUN i. $\sigma^{2}$ is the noise power. Thus, the uplink transmission delay of the sensor j in WBAN i can be described as:

(7) $$T_{ij}^{\mathrm{SN}}=\frac{d_{ij}{}^{\mathrm{SN}}}{r_{ij}^{\mathrm{SN}}}$$

where $d_{ij}^{\mathrm{SN}}$ depicts the size of the medical monitoring data packet generated by the sensor j in WBAN i.

In this paper, it is assumed that the scheduling period of the WBAN is $T^{\mathrm{WBAN}}$, which contains T data transmission time slots. The time slot set can be denoted by T={0,1,…,T−1}. Assuming that the length of each time slot is L, the size of the slot block required for the sensor j in WBAN i to transmit its data packet is:

(8) $$N_{ij}^{\mathrm{SN}}=\left\lceil \frac{T_{ij}^{\mathrm{SN}}}{L}\right\rceil$$

The GUN i first gathers all medical packets in the WBAN i at the end of the scheduling period and then selects the appropriate ES for data offloading. The vector $u=\{u_{i,j}(t)|i\in N,j\in S,t\in T\}$ is used to represent the slot allocation, where $u_{i,j}(t)=1$ depicts that the sensor j in WBAN i occupies the t-th slot. Therefore, the size of the collected packet can be expressed as:

(9) $$d_{i}^{\mathrm{GUN}}=\sum_{j=1}^{S}Y(\sum_{t=1}^{T}u_{i,j}(t)>0)d_{ij}^{\mathrm{SN}}$$

where Y(⋅) is an indicator function, and Y(g)=1 if g>0; if otherwise, Y(g)=0.

To monitor the health status of patients more comprehensively, multiple types of sensors are utilized in the WBANs to evaluate various physiological indicators. For example, electrocardiographic (ECG) sensors monitor heart rate and blood pressure, while electroencephalographic (EEG) sensors monitor cortical activities [25]. To reflect the health criticality of the patients from a medical perspective, the medical urgency of the packets is also considered in this paper. It is assumed that all medical monitoring packets are classified into K categories according to the medical urgency, denoted by K={1,2,…,K}. Let $x_{ijk}=1$ represent that the medical monitoring packet belongs to the k-th class generated by the sensor j in WBAN i, and $x_{ijk}=0$ represents other cases. Therefore, the medical urgency of the medical packet collected by GUN i can be expressed as:

(10) $$B_{i}=\sum_{j=1}^{S}Y(\sum_{t=1}^{T}u_{i,j}(t)>0)\sum_{k=1}^{K}x_{ijk}\delta_{k}$$

where $\delta_{k}$ represents the coefficient of medical urgency. The higher value of the coefficient represents a higher level of medical urgency.

### 3.3. Communication Model for the ECN

The MC may be made aware of the precise participation willingness of each ES after they have all signed the contracts. The MC will broadcast the contract information of all ESs to the GUNs, and after that, ESs need to provide service according to the contracts they signed.

Due to the limitations of GUNs’ computing resources and energy consumption, as well as the integrity of medical monitoring data, partial data offloading was not considered in this article. To enhance spectrum efficiency, the NOMA technology is adopted for data transmission in the ECN. The data offloading rate between the GUN i and the ES m can be expressed as [26]:

(11) $$r_{im}^{\mathrm{GUN}}=q_{m}\log_{2}(1+\frac{p_{i}^{\mathrm{GUN}}h_{im}^{\mathrm{GUN}}}{\sum_{k\in N\backslash \{i\}:a_{km}=1}p_{k}^{\mathrm{GUN}}h_{km}^{\mathrm{GUN}}+\sigma^{2}})$$

where $p_{i}^{\mathrm{GUN}}$ represents the transmission power of GUN i, and $h_{im}^{\mathrm{GUN}}$ represents the channel gain between the GUN i and the ES m.

According to Equations (9) and (11), the delay and energy consumption for the GUN i transmitting medical monitoring packets to the ES m can be written, respectively, as:

(12) $$T_{im}^{\mathrm{tran}}=\frac{d_{i}^{\mathrm{GUN}}}{r_{im}^{\mathrm{GUN}}}$$

(13) $$E_{im}^{\mathrm{tran}}=p_{i}^{\mathrm{GUN}}T_{im}^{\mathrm{tran}}$$

After ESs receive the collected packets, the computing resources are allocated according to the level of medical urgency. The computing resources allocated to the packets provided by the GUN i can be expressed as:

(14) $$F_{im}=F_{s}*\frac{B_{i}}{\sum_{k=1}^{N}a_{km}B_{k}}$$

where $F_{s}$ represents the total computing resources of ESs. $a=\{a_{im}|i\in N,m\in M\}$ is the offloading strategy of all GUNs, where $a_{im}=1$ represents the GUN i selecting the ES m to offload medical data packets. The computing delay of the packets from the GUN i is:

(15) $$T_{im}^{\mathrm{com}}=\frac{f_{i}d_{i}^{\mathrm{GUN}}}{F_{im}}$$

where $f_{i}$ represents the computation workload/intensity (in CPU cycles per bit), i.e., the number of CPU cycles for ES i to process 1 bit data.

### 3.4. System Utility

The quality of experience (QoE) can be described by the timeliness of the medical data for updates and the energy consumption in the intelligent telemedicine system. Thus, the cost of monitoring data for patient i can be described as the weighted sum of the AoI, transmission time, computing time at the ES, and energy consumption, which can be expressed as:

(16) $$C_{i}^{\mathrm{data}}=\beta^{T}\sum_{m=1}^{M}a_{im}(\sum_{j=1}^{S}A_{ij}(t)+T_{im}^{\mathrm{tran}}+T_{im}^{\mathrm{com}})+\beta^{E}\sum_{m^{\prime }=1}^{M}a_{im^{\prime }}(E_{im^{\prime }}^{\mathrm{tran}})$$

where $\beta^{T}$ and $\beta^{E}$ are the weight factors for the time parameters and energy consumption, and $\beta^{T}+\beta^{E}=1$. As each sensor in the WBANs uses TDMA technology to access the channel and the wireless resource is shared by all sensors, the data transmission for each sensor would lead to relatively fixed energy consumption. Thus, the transmission energy consumption of all sensors within the WBAN is not considered in Equation (16). Theoretically, the time for the monitoring data to be transmitted to the MC should include the scheduling period of the WBAN, the transmission delay of the medical packets offloaded to the ESs, the processing delay of the packets for the ESs, and the time for the ESs to upload the processing results to the MC. Since the size of packets after being processed by the ESs is usually small enough, the time for the ESs to upload analysis results to the MC is ignored in this paper.

In order to enhance the utility of the MC while meeting the needs of the patients, the system utility can be defined as the difference between the utility of the MC and the system cost:

(17) $$W=U_{\mathrm{MC}}-\sum_{i=1}^{N}C_{i}^{\mathrm{data}}$$

## 4. Resource Allocation and Data Offloading Based on Optimal Contracts

Our goal is to maximize the system utility in an intelligent telemedicine system, and the optimization problem can be modeled as follows:

(18) $$\underset{(q,\pi ),u,a}{\max}W=U_{\mathrm{MC}}-\sum_{i=1}^{N}\{\beta^{T}\sum_{m=1}^{M}a_{im}(\sum_{j=1}^{S}A_{ij}(t)+T_{im}^{\mathrm{tran}}+T_{im}^{\mathrm{com}})+\beta^{E}\sum_{m^{\prime }=1}^{M}a_{im^{\prime }}(E_{im^{\prime }}^{\mathrm{tran}})\}$$

Subject to the following constraints:

(19) $$a_{im}\in \{0,1\},\forall i\in N,m\in M$$

(20) $$u_{i,j}(t)\in \{0,1\},\forall i\in N,j\in S,t\in T$$

(21) $$\sum_{j=1}^{S}N_{ij}^{\mathrm{SN}}\leq T,\forall i\in N$$

(22) $$\sum_{m=1}^{M}a_{im}=1,\forall i\in N$$

(23) $$\sum_{i=1}^{N}a_{im}\leq N^{\mathrm{SL}}$$

where (q,π) is the set of the optimal contracts provided by the MC for all types of ESs. u and a represent the slot allocation strategy for the WBAN and the data offloading strategy for the ECN, respectively. Constraint Equations (19) and (20) indicate that the slot allocation variable and the offloading strategy variable are binary variables. Constraint Equation (21) guarantees that the allocated slots cannot exceed the threshold T. Constraint Equation (22) shows that each GUN can only choose one ES. The constraint of the service limitation of ESs is shown in Equation (23). Based on Equation (11), we can observe that when a GUN’s collected medical monitoring packet is offloaded to the ES, its cost depends both on the offloading strategies of other GUNs and its own. Moreover, if too many GUNs choose the same ES for auxiliary computing, the communication resources provided by the ES need to be shared by multiple GUNs. These GUNs will suffer from severe transmission interference to the extent that the data offloading rate is affected, which eventually generates a large system cost. In addition, too many GUNs accessing the same ES will bring additional equipment access costs and affect the willingness of the ES to participate in the cooperation. In this case, it is necessary to limit the number of GUNs for ES services.

Since the constraints and optimization variables in the optimization problem Equation (18) are interdependent, we can rewrite it in the following form to decouple them:

(24) $$\underset{(q,\pi ),u,a}{\max}W=U_{\mathrm{MC}}-\sum_{i=1}^{N}C_{i}^{\mathrm{data}}=U_{\mathrm{MC}}-C^{\mathrm{WBAN}}-C^{\mathrm{ECN}}$$

where:

(25) $$C^{\mathrm{WBAN}}=\beta^{T}\sum_{i=1}^{N}\sum_{j=1}^{S}A_{ij}(t)$$

(26) $$C^{\mathrm{ECN}}=\sum_{i=1}^{N}\{\beta^{T}\sum_{m=1}^{M}a_{im}(T_{im}^{\mathrm{tran}}+T_{im}^{\mathrm{com}})+\beta^{E}\sum_{m^{\prime }=1}^{M}a_{im^{\prime }}(E_{im^{\prime }}^{\mathrm{tran}})\}$$

Based on Equation (24), the goal of maximizing the system utility can be achieved by maximizing the MC utility and minimizing the system cost, which consists of the cost for the WBAN $C^{\mathrm{WBAN}}$ and the cost for the ECN $C^{\mathrm{ECN}}$. Specifically, maximizing the utility of the MC corresponds to the optimal contract formulation problem between the MC and the ESs, minimizing the cost for the WBAN corresponds to the slot allocation problem, and minimizing the cost for the ECN corresponds to the data offloading problem.

### 4.1. Optimal Contract Formulation between MC and ESs

Taking the first term in Equation (24) as the objective function, the maximization problem of the MC’s utility can be written as:

(27) $$\underset{\left(q,\pi \right)}{\max}U_{\mathrm{MC}}=\sum_{i=1}^{I}\xi_{i}[\alpha q_{i}(\theta_{i})-\pi_{i}(\theta_{i})]$$

Subject to the following constraints:

(28) $$U_{\mathrm{ES}}(i)=\theta_{i}v(\pi_{i}(\theta_{i}))-cq_{i}(\theta_{i})\geq 0,\forall i\in I$$

(29) $$\theta_{i}v(\pi_{i}(\theta_{i}))-cq_{i}(\theta_{i})\geq \theta_{i}v(\pi_{j}(\theta_{j}))-cq_{j}(\theta_{j}),\forall i,j\in I,i\neq j$$

where Equations (28) and (29) are the individual rationality (IR) conditions and incentive compatibility (IC) conditions for the feasible contracts based on contract theory [27]. For the IR condition, it ensures that ESs select contracts that maintain the non-negativity of their own utility. The IC condition ensures that the maximum benefit is obtained if and only if the ESs select a contract that matches their participation willingness.

Since the participation willingness of ESs is unknown to the MC, there are total I IR constraints and I(I−1) IC constraints in the optimization problem Equation (27). In order to facilitate the solution, we first simplify these constraints.

**Lemma** **1.** *For any feasible contract* $\left(q_{i}(\theta_{i}),\pi_{i}(\theta_{i})\right)$*, there is* $\pi_{i}(\theta_{i})>\pi_{j}(\theta_{j})$ *if and only if* $\theta_{i}>\theta_{j}$.

**Proof** **of** **Lemma** **1.** See Appendix A. □

**Lemma** **2.** *The feasible contract* $\left(q_{i}(\theta_{i}),\pi_{i}(\theta_{i})\right)$ *satisfies* $\pi_{i}(\theta_{i})>\pi_{j}(\theta_{j})$ *if and only if* $q_{i}(\theta_{i})>q_{j}(\theta_{j})$.

**Proof** **of** **Lemma** **2.** See Appendix B. □

**Corollary** **1.** *The feasible contract* $\left(q_{i}(\theta_{i}),\pi_{i}(\theta_{i})\right)$ *satisfies* $\pi_{i}(\theta_{i})=\pi_{j}(\theta_{j})$ *if and only if* $q_{i}(\theta_{i})=q_{j}(\theta_{j})$.

Based on Lemma 1, Lemma 2, and the Corollary 1, to make the optimal contract satisfy the condition that ESs with higher participation willingness will provide more communication resources while obtaining higher rewards from the MC, the following constraint can be obtained:

(30) $$0\leq \pi_{1}\leq \ldots \leq \pi_{I}$$

**Lemma** **3.** *If the ESs with participation willingness* $\theta_{1}$ *satisfies the IR condition, all ESs will automatically meet the IR condition*.

**Proof** **of** **Lemma** **3.** See Appendix C. □

Based on Lemma 3, I IR conditions for a feasible contract can be simplified to a single IR condition as follows:

(31) $$\theta_{1}v(\pi_{1}(\theta_{1}))-cq_{1}(\theta_{1})\geq 0$$

To simplify the IC constraints in Equation (27), we first define that the downward IC (DIC) condition is between the ES with participation willingness $\theta_{i+1}$ and the ES with participation willingness $\theta_{i^{\prime }}(i^{\prime }\in \{1,\ldots ,i\})$ while the upward IC (UIC) condition is between the ES with participation willingness $\theta_{i}$ and the ES with participation willingness $\theta_{i^{\prime }}(i^{\prime }\in \{i+1,\ldots ,I\})$ [28]. The local DIC (LDIC) condition is a special case of the DIC condition, i.e., the condition between the ES with participation willingness $\theta_{i}$ and the ES with participation willingness $\theta_{i-1}$. The local UIC condition is a special case of the UIC condition, i.e., the condition between the ES with participation willingness $\theta_{i}$ and the ES with participation willingness $\theta_{i+1}$.

**Lemma** **4.** *The IC conditions can be simplified to LDIC conditions and LUIC conditions, i.e.,*

(32) $$\theta_{i}v(\pi_{i}(\theta_{i}))-cq_{i}(\theta_{i})\geq \theta_{i}v(\pi_{i-1}(\theta_{i-1}))-cq_{i-1}(\theta_{i-1}),\forall i\in \{2,3,\ldots ,I\}\;$$

(33) $$\theta_{i}v(\pi_{i}(\theta_{i}))-cq_{i}(\theta_{i})\geq \theta_{i}v(\pi_{i+1}(\theta_{i+1}))-cq_{i+1}(\theta_{i+1}),\forall i\in \{1,2,\ldots ,I-1\}\;$$

**Proof** **of** **Lemma** **4.** See Appendix D. □

Thus, the optimization problem in Equation (27) can be simplified as:

(34) $$\underset{\left(q,\pi \right)}{\max}U_{\mathrm{MC}}=\sum_{i=1}^{I}\xi_{i}[\alpha q_{i}(\theta_{i})-\pi_{i}(\theta_{i})]$$

Subject to the following constraints:

(35) $$\theta_{1}v(\pi_{1}(\theta_{1}))-cq_{1}(\theta_{1})\geq 0$$

(36) $$\theta_{i}v(\pi_{i}(\theta_{i}))-cq_{i}(\theta_{i})\geq \theta_{i}v(\pi_{i-1}(\theta_{i-1}))-cq_{i-1}(\theta_{i-1})$$

(37) $$0\leq \pi_{1}\leq \ldots \leq \pi_{I}$$

The difficulty of solving the optimization problem after simplifying the redundant constraints is significantly reduced. Iterating over the constraint Equations (35) and (36), there will be:

(38) $$q_{i}=\frac{1}{c}\left\{\theta_{i}v(\pi_{i}(\theta_{i}))+\sum_{j=2}^{i}\left(\theta_{j-1}-\theta_{j})v(\pi_{j-1}(\theta_{j-1})\right)\right\}$$

By substituting Equation (38) into Equation (34), the objective function can be expressed as:

(39) $$\underset{\pi }{\max}U_{\mathrm{MC}}=\sum_{i=1}^{I}\xi_{i}\left\{\frac{\alpha }{c}\left[\theta_{i}v(\pi_{i}(\theta_{i}))+\sum_{j=2}^{i}\left(\theta_{j-1}-\theta_{j})v(\pi_{j-1}(\theta_{j-1})\right)\right]-\pi_{i}(\theta_{i})\right\}$$

The optimal values $\pi^{*}$ can be obtained by solving the problem Equation (39). Then, the optimal values $q^{*}$ will be achieved by substituting $\pi^{*}$ into Equation (34). The formulation of the optimal contract based on contract theory (CT) is shown in Algorithm 1.
**Algorithm 1** The formulation of the optimal contract based on contract theory1: Obtain the constraints of the feasible contract based on IR condition and IC condition 2: Obtain the constraint Equation (30) based on Lemma1, Lemma 2, and the corollary 3: Simplify the I IR conditions based on Lemma 3 4: Simplify the I(I−1) IC conditions based on Lemma 4 5: Obtain the simplified optimization problem (34) $6:\;\mathrm{Obtain}\;\mathrm{the}\;\mathrm{optimal}\;\mathrm{values}\;\pi^{*}$ based on (39) $7:\;\mathrm{Obtain}\;\mathrm{the}\;\mathrm{optimal}\;\mathrm{values}\;q^{*}$$\;\mathrm{based}\;\mathrm{on}\;\pi^{*}$ by solving the (34) **Output:** 8: The optimal contract (q,π)

### 4.2. Slot Allocation for the WBAN Based on Cooperative Game

Taking the second term in Equation (24) as the objective function, the slot allocation problem for the WBAN can be written as:

(40) $$\underset{u}{\min}C^{\mathrm{WBAN}}=\sum_{i=1}^{N}\sum_{j=1}^{S}A_{ij}(t)$$

Subject to the following constraints:

(41) $$u_{i,j}(t)\in \{0,1\},\forall i\in N,j\in S,t\in T$$

(42) $$\sum_{j=1}^{S}N_{ij}^{\mathrm{SN}}\leq T,\forall i\in N$$

The weight factor $\beta^{T}$ is a constant that is not considered in the optimization problem (40). Thus, the minimization of the cost for the WBAN is equivalent to the AoI minimization problem for each sensor. The cost of the sensor j in the WBAN i can be expressed as:

(43) $$C_{ij}^{\mathrm{SN}}=A_{ij}(t)$$

Moreover, to facilitate the analysis, the opposite of the cost is used as the utility function of the sensors and the WBAN:

(44) $$U_{ij}^{\mathrm{SN}}=-C_{ij}^{\mathrm{SN}}$$

(45) $$U_{i}^{\mathrm{WBAN}}=-\sum_{j=1}^{S}C_{ij}^{\mathrm{SN}}$$

where $U_{ij}^{\mathrm{SN}}$ is the utility of the sensor j in WBAN i, while $U_{i}^{\mathrm{WBAN}}$ is the utility of WBAN i.

All sensors in the WBAN serve collaboratively, and any data obsolescence can cause a dramatic increase in the cost of the WBAN. Therefore, sensors are motivated to cooperate to ensure the overall utility of the WBAN. We describe the slot allocation problem for the WBAN as a cooperative game problem. In the cooperative game, each sensor competes for channel resources by adjusting its slot occupation, but each sensor aims to maximize the utility of the corresponding WBAN on the basis of ensuring its own utility.

The cooperative game problem for WBAN can be formulated as:

(46) $$\Omega_{0}^{\mathrm{WBAN}}=\left\{S,{\left\{u_{i,j}\right\}}_{j\in S},\{U_{ij}^{\mathrm{SN}}{\left(u_{i,j},u_{i,-j}\right\}}_{j\in S}\right\}$$

where ${\left\{U_{ij}^{\mathrm{SN}}(u_{i,j},u_{i,-j})\right\}}_{j\in S}$ is the set of the sensors’ utility for feasible slot allocation. $u_{i,j}$ represents the slots allocated to the sensor j in WBAN i, while $u_{i,-j}$ represents the slots allocated to the other sensors in WBAN i.

As mentioned earlier, S sensors in each WBAN participate in the game, and each sensor generates monitoring data and transmits it as soon as the slots are allocated. Therefore, the AoI of the sensors which are allocated slots is equal to the transmission slots plus the remaining time of the WBAN scheduling. Although the sensors aim to minimize the cost for the WBAN, there are still some limited cases. All sensors achieve the maximum AoI if there is no competition for slots during the WBAN scheduling period. Therefore, this case can be considered as the lower bound of the feasible slot allocation, where the corresponding utility of the WBAN is minimized. The upper bound of the slot allocation is that the AoI of all sensors does not change during the whole scheduling period, where the corresponding utility of the WBAN is maximized. Thus, the feasible minimum and the unreachable maximum utility of the sensor are:

(47) $${\tilde{U}}_{ij}^{\min}=-(A_{ij}(t)+T^{\mathrm{WBAN}})$$

(48) $${\tilde{U}}_{ij}^{\max}=-A_{ij}(t)$$

The objective of the cooperative game for the WBAN is to maximize its utility. Thus, the game $\Omega_{0}^{\mathrm{WBAN}}$ can be equivalent to:

(49) $$\Omega_{1}^{\mathrm{WBAN}}=\left\{S,{\left\{u_{i,j}\right\}}_{j\in S},{\left\{U_{i}^{\mathrm{WBAN}}(u_{i,j},u_{i,-j})\right\}}_{j\in S}\right\}$$

where the optimal slot allocation is:

(50) $$u_{i,j}^{*}\in \arg\max U_{i}^{\mathrm{WBAN}}(u_{i,j},u_{i,-j})$$

**Theorem** **1.** *The feasible utility set of the WBAN’s cooperative game *$\Omega_{1}^{\mathrm{WBAN}}$ *has a maximum value.*

**Proof** **of** **Theorem** **1.** Based on Equations (47) and (48), the set of all sensors’ utility can be expressed as:

(51) $$U_{i}^{\mathrm{SN}}=\{U_{ij}^{\mathrm{SN}}|{\tilde{U}}_{ij}^{\min}\leq U_{ij}^{\mathrm{SN}}<{\tilde{U}}_{ij}^{\max},\forall j\in S\}$$

Thus, the set of the feasible utility for the WBAN can be expressed as:

(52) $$U_{i}^{\mathrm{WBAN}}=\left\{U_{i}^{\mathrm{WBAN}}|\sum_{j=1}^{S}{\tilde{U}}_{ij}^{\min}\leq U_{i}^{\mathrm{WBNA}}<\sum_{j=1}^{S}{\tilde{U}}_{ij}^{\max}\right\}$$

The set of the feasible WBAN’s utility has a maximum value if and only if the cardinality of $U_{i}^{\mathrm{WBAN}}$ is less than infinity. From Equations (47) and (48), it can be seen that the utility values of the sensors vary discretely between the minimum and the maximum with the different slot allocations. Thus, the feasible utility set of the WBAN is limited, and the maximum value exists. □

A slot allocation algorithm based on collaborative game is proposed to obtain the optimal solution for the optimization problem of Equation (40). The details are shown in Algorithm 2.
**Algorithm 2.** Slot allocation based on collaborative game**Input:** 1: The initial maximum utility for every WBAN: $U_{\max}^{\mathrm{WBAN}}=\sum_{j=1}^{S}{\tilde{U}}_{ij}^{\min}$ **Initialization:** 2: Slot allocation strategy u$:\;u_{i,j}(t)=0$ $3:\;\mathrm{Remaining}\;\mathrm{unallocated}\;\mathrm{slots}\;\mathrm{in}\;\mathrm{reverse}\;\mathrm{order}\;T_{r}$$:\;T_{r}=\{T,T-1,\ldots ,1\}$ **Optimization:** 4: for each WBAN i, i∈N **do** 5:    for each sensor j, j∈S **do** $6:\;\;\;\;\;\;\mathrm{Each}\;\mathrm{sensor}\;\mathrm{occupies}\;\mathrm{the}\;\mathrm{slots}\;\mathrm{in}\;\mathrm{reverse}\;\mathrm{order}\;\mathrm{of}\;\mathrm{the}\;\mathrm{scheduling}\;\mathrm{period}\;\mathrm{of}\;\mathrm{the}\;\mathrm{WBAN}\;\mathrm{and}\;\mathrm{computes}\;\mathrm{the}\;\mathrm{utility}\;U_{i}^{\mathrm{WBAN}}(u_{i,j})$ based on (33) $7:\;\;\;\;\;\;\;\;\mathrm{if}\;U_{i}^{\mathrm{WBAN}}(u_{i,j})>U_{\max}^{\mathrm{WBAN}}$ **then** $8:\;\;\;\;\;\;\;\;\;\;u\leftarrow u_{i,j}$ $9:\;\;\;\;\;\;\;\;\;\;\mathrm{Update}\;\mathrm{the}\;\mathrm{maximum}\;\mathrm{system}\;\mathrm{utility}\;\mathrm{to}\;U_{i}^{\mathrm{WBAN}}(u_{i,j})$ 10:             Update the unallocated slots in reverse order by removing the allocated slots 11:         **end if** 12:   **end** 13: **end** **Output:** $14:\;\mathrm{Optimal}\;\mathrm{time}\;\mathrm{slot}\;\mathrm{allocation}\;\mathrm{strategy}:\;u^{*}$


### 4.3. Data Offloading for the ECN Based on Bilateral Matching Game

Taking the third term in Equation (24) as the objective function, the data offloading problem for the ECN can be written as:

(53) $$\underset{a}{\min}C^{\mathrm{ECN}}=\sum_{i=1}^{N}\sum_{m=1}^{M}a_{im}\{\beta^{A}(T_{im}^{\mathrm{tran}}+T_{im}^{\mathrm{com}})+\beta^{E}E_{im}^{\mathrm{tran}}\}$$

Subject to the following constraints:

(54) $$a_{im}\in \{0,1\},\forall i\in N,m\in M$$

(55) $$\sum_{m=1}^{M}a_{im}=1,\forall i\in N$$

(56) $$\sum_{i=1}^{N}a_{im}\leq N^{\mathrm{SL}}$$

Within the ECN, the interaction between the GUNs and the ESs in the optimization problem of Equation (53) involves 0–1 variables; thus, the bipartite matching game model was introduced to solve the problem.

**Definition** **1.** *For the disjoint sets of GUNs and ESs, bipartite matching needs to satisfy the following conditions: (1)* μ(i)∈M,μ(m)∈N∪∅*; (2)* $|\mu (i)|=1,|\mu (m)|\leq N^{\mathrm{SL}}$*; where* i∈N *and* m∈M.

In order to measure the impact of the GUN’s selection of different ESs on the cost for the ECN, the cost generated by the GUN’s selection is defined as the selection cost, which is calculated as follows:

(57) $$C_{im}^{S}=\beta^{A}(T_{im}^{\mathrm{tran}}+T_{im}^{\mathrm{com}})+\beta^{E}E_{im}^{\mathrm{tran}}$$

where the selection cost of GUNs cannot be accurately calculated because we cannot get the inter-user interference and the division of the computation resources before the matching result. Initially, we assume that there is no inter-user interference and the computation resources of each ES are equally divided based on the service limitation.

**Preference List of the GUNs**: For GUNs, every GUN wants to select the ES at the lowest cost. Thus, for GUN i, i∈N, its preference over ES m, m∈M can be expressed as:

(58) $$PL_{\mathrm{GUN}}(i,m)=C_{im}^{S}$$

The preference list can be acquired by sorting every row of N×M matrix $PL_{\mathrm{GUN}}$ in ascending order.

**Preference List of the ESs**: From a system perspective, we are attempting to minimize the cost of the ECN caused by the different choices of the ESs as much as possible. Therefore, each ES prefers to match with the GUNs whose selection cost is much lower. For ES m, m∈M, its preference over GUN i, i∈N can be expressed as:

(59) $$PL_{\mathrm{ES}}(m,i)=C_{im}^{S}$$

The preference list can be acquired by sorting every row of M×N matrix $PL_{\mathrm{ES}}$ in ascending order.

The preference lists offered in Equations (58) and (59) are initial values that are always modified when a stable equilibrium cannot be reached.

The exact preference list is not available initially, and the preference lists between different GUNs affect each other during the matching process. Therefore, there is an externality in the bilateral matching model studied in this paper. In order to ensure the stability of the final matching result, the matching object of the established match result can be exchanged based on the gain matching, which can improve the cost of the ECN progressively.

**Definition** **2.** *For the matching pair* (i,m) *of the matching result, if change the matching object of* i *to* $m^{\prime }$*, where* i∈N *and* $m,m^{\prime }\in M$ *will get the finite gain, the gain matching* $\mu_{i}^{mm^{\prime }}$ *can be defined as* $\mu_{i}^{mm^{\prime }}=\{\mu \backslash (i,m)\}\cup (i,m^{\prime })$.

Finite gain signifies that by swapping out the matching objects, the participants can lower the cost to some extent. Gain matching provides the GUNs with the opportunity to change the matching objects, but gain matching only takes effect after obtaining the permission of the ESs. For ESs, only a gain matching process that can reduce the cost for the ECN will be allowed.

**Definition** **3.** *If there is no blocking pair in the matching result* μ*, it can be called stable matching.*

**Definition** **4.** *If the matching pair* (i,m) *satisfies the following two conditions: (1) GUN* i *is unmatched or there is a gain matching object* m *of GUN* i*; (2) there is a gain matching object* i of ES m; the (i,m) *can be called a blocking pair.*

Data offloading strategy based on bilateral matching game is shown in Algorithm 3.
**Algorithm 3** Data offloading strategy based on bilateral matching game**Input:** 1: The set of GUNs: N 2: The set of ESs: M 3: The initial match result μ: μ=∅ $4:\;\mathrm{Unmatched}\;\mathrm{GUNs}\;N_{u}$$:\;N_{u}=N$ **Initialization:** $5:\;\mathrm{Compute}\;\mathrm{the}\;\mathrm{initial}\;\mathrm{preference}\;\mathrm{list}\;PL_{\mathrm{GUN}}$$\;\mathrm{and}\;PL_{\mathrm{ES}}$ under the assumptions that there is no inter-user interference and the computing resources on ESs are equally divided according to the service limitation $6:\;\mathrm{while}\;N_{u}!=\emptyset$ **do** 7:     Unmatched GUNs send service requests to the ESs according to their preference list $8:\;\;\;\;\mathrm{The}\;\mathrm{ESs}\;\mathrm{receives}\;\mathrm{the}\;\mathrm{top}\;N^{\mathrm{SL}}$ GUNs in their preference list and refuses other GUNs 9:     The rejected GUNs remove the ESs which reject them from their preference list 10: **end** 11: Obtain the match result μ **Optimization:** $12:\;\mathrm{Compute}\;\mathrm{the}\;\mathrm{preference}\;\mathrm{list}\;PL_{\mathrm{GUN}}$$\;\mathrm{and}\;PL_{\mathrm{ES}}$ based on (43) and (44) $13:\;\mathrm{while}\;\mathrm{there}\;\mathrm{exists}\;\mathrm{the}\;\mathrm{gain}\;\mathrm{matching}:\;\mu_{i}^{mm^{\prime }}=\{\mu \backslash (i,m)\}\cup (i,m^{\prime })$ in μ **do** 14:    GUN i$\;\mathrm{send}\;a\;\mathrm{service}\;\mathrm{request}\;\mathrm{to}\;\mathrm{ES}\;m^{\prime }$ $15:\;\;\;\;\mathrm{if}\;|\mu (m^{\prime })|\leq N^{\mathrm{SL}}$ **then** $16:\;\;\;\;\;\;\;\;\{\mu \backslash (i,m)\}\cup (i,m^{\prime })$ 17:     **else** $18:\;\;\;\;\;\;\;\;\mathrm{ES}\;m^{\prime }$$\;\mathrm{receives}\;\mathrm{temporarily}\;\mathrm{the}\;\mathrm{top}\;N^{\mathrm{SL}}$ GUNs and refuses other GUNs 19:     **endif** $20:\;\;\;\mathrm{Recalculate}\;\mathrm{the}\;\mathrm{preference}\;\mathrm{list}\;PL_{\mathrm{GUN}}$$\;\mathrm{and}\;PL_{\mathrm{ES}}$ 21: **end while** **Output:** $22:\;\mathrm{Stable}\;\mathrm{match}\;\mathrm{result}:\;\mu_{s}$ 23: Obtain the offloading strategy a$\;\mathrm{based}\;\mathrm{on}\;\mu_{s}$


### 4.4. Joint Strategy for Resource Allocation and Data Offloading Based on the Optimal Contracts

In summary, the system utility maximization problem is split into two parts for solving: one is the utility maximization problem of the MC, i.e., the optimal contract formulation problem between the MC and the ESs, and the other is the system cost minimization problem. Specifically, the system cost minimization problem is composed of the slot allocation problem for the WBAN to minimize the cost for the WBAN and the data offloading problem for the ECN to minimize the cost for the ECN. The process of resource allocation and data offloading needs to be performed on the basis of the optimal contract signed by the ESs. Thus, a joint strategy for resource allocation and data offloading (JSRADO) based on the optimal contract which is composed of Algorithm 2 and Algorithm 3 is proposed, and the details are shown in Algorithm 4:
**Algorithm 4** Joint strategy for resource allocation and data offloading**Input:** 1: The optimal contracts: (q,π) obtained by the CT strategy (**Algorithm 1**) **Optimization:** 2: for each GUN i∈N **do** 3:   Allocated slot resources based on **Algorithm 2** 4: **end for** 5: GUNs broadcast the information of their collected medical monitoring data packets 6: ESs broadcast the information of communication resources 7: All GUNs execute **Algorithm 3** in parallel **Output:** 8: The slot allocation profile u and the data offloading strategy a


## 5. Simulation Results and Analysis

In this section, the proposed strategy for resource allocation and data offloading is simulated and verified. In the intelligent telemedicine scenario, there are five external auxiliary ESs in a circular area with a 250 m radius, and the number of the patients is N=30. It is assumed that various medical sensors are distributed in a star-shaped architecture centered on the GUNs. Based on [29,30], the channel gain is set as $h_{i,m}=l_{i,m}^{-\eta }$, where $l_{i,m}$ is the distance between the GUN i and its accessed ES m, and η denotes the path loss factor. Additionally, we considered eight different levels of medical urgency, i.e., K=8 based on IEEE 802.15.6 [5]. The other simulation parameters in this paper are shown in Table 2.

In order to verify the effectiveness of the proposed strategy composed of the CT algorithm and the JSRADO algorithm, the simulation results will be compared with the following algorithms, respectively:

- The contract mechanism based on complete information (CI) [31]: the algorithm is based on the ideal case where the MC can be informed of the participation willingness of each ES in advance, in which case the ESs cannot misrepresent the information in order to obtain additional benefits;
- Stackelberg game incentive mechanism under asymmetry information scenario (SGA) [32]: the MC as a leader makes different contracts according to the participation willingness of ESs, and the ES as a follower decides the communication resources to provide according to the rewards of the contract;
- Minimum Distance (MD) [33]: all gathered monitoring data in the GUNs are offloaded to the nearest ES, which can reduce path loss to the most extent;
- The resident-oriented Gale–Shapley algorithm (RGS) [22]: the GUNs select ESs for access according to the preference list of the GUNs, and ESs admit GUNs in the order of the preference list of the ESs;
- Decentralized game theoretic approach for health monitoring (DIGTAL) [30]: a decentralized game theoretic approach is used to model the process of data offloading in order to minimize the system cost.

**Figure 2:**
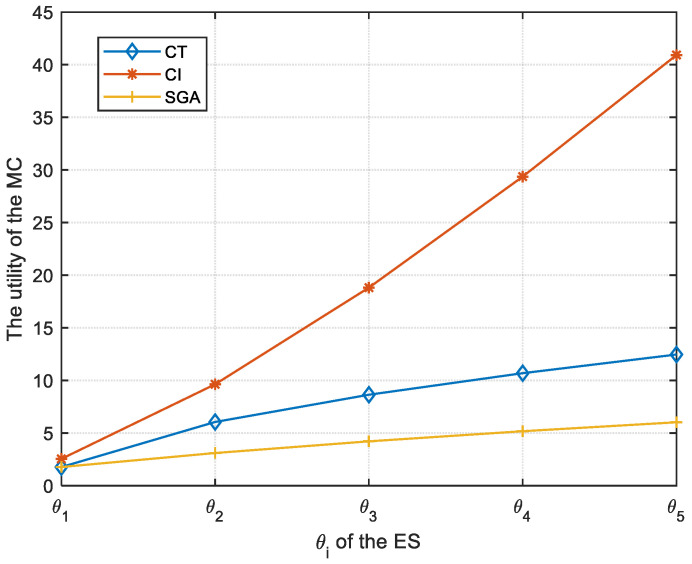
The utility of the MC varies with $\theta_{i}$.

Figure 2 shows the comparison of three algorithms in terms of the utility of the MC. It can be observed that the utility of the MC increases with a stronger participation willingness of the ES. From Figure 2, the utilities of the MC for the CI algorithm are larger than that by using the CT or SGA algorithm. However, the CI algorithm is carried out based on the fact that the MC is aware of the participation willingness of all ESs in advance, which is hard to achieve in practical applications. If the contract is signed between the MC and the ESs by using the CT or the SGA algorithm, it is not necessary for the MC to know all participation willingness of the ESs. Therefore, the CT and the SGA algorithm are more suitable for practical applications to enhance the utility of the MC. Moreover, the utility of the MC using the CT algorithm is larger than that by adopting the SGA algorithm with the same participation willingness of the ES.

**Figure 3:**
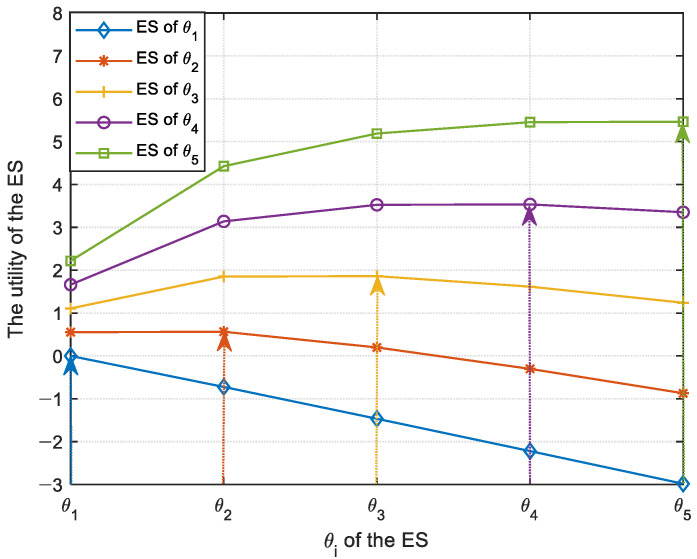
The maximum utility of the ES varies with $\theta_{i}$.

In Figure 3, the optimal contract between the MC and the ES is evaluated by verifying the IR and IC conditions. The maximum utility value of the ES will increase if the value of $\theta_{i}$ becomes larger, which means that the increase in the participation willingness can enhance the utility of the ES. It can be seen from Figure 3 that the IC condition is verified by the fact that the maximum utility of the ES is obtained only if the contract matches its participation willingness $\theta_{i}$, in which case the utility of the ES remains non-negative, verifying the IR condition as well.

**Figure 4:**
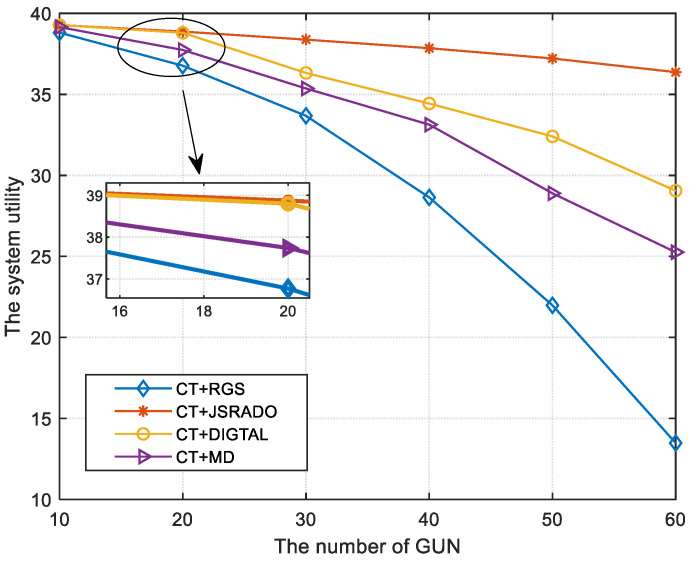
The system utility with different numbers of GUNs.

Figure 4 illustrates the trend of system utility as the number of GUNs is changed. The system utility is reduced if the number of GUNs becomes larger, which means that more participants compete for the communication resources provided by the ESs, and more participants need to share ESs’ computing resources. Therefore, it will bring a greater transmission delay and energy loss for each GUN, which indicates that the system cost will become larger. Moreover, the JSRADO algorithm can obtain a higher system utility compared to the RGS algorithm, the MD algorithm, and the DIGTAL algorithm by providing matched communication and computing resources for the GUNs. If the number of the GUNs becomes smaller, which means that the communication resources provided by the ESs are rich enough to satisfy the requirements for all GUNs, the difference of the system utility for the proposed JSRADO algorithm and the compared algorithms will become smaller.

**Figure 5:**
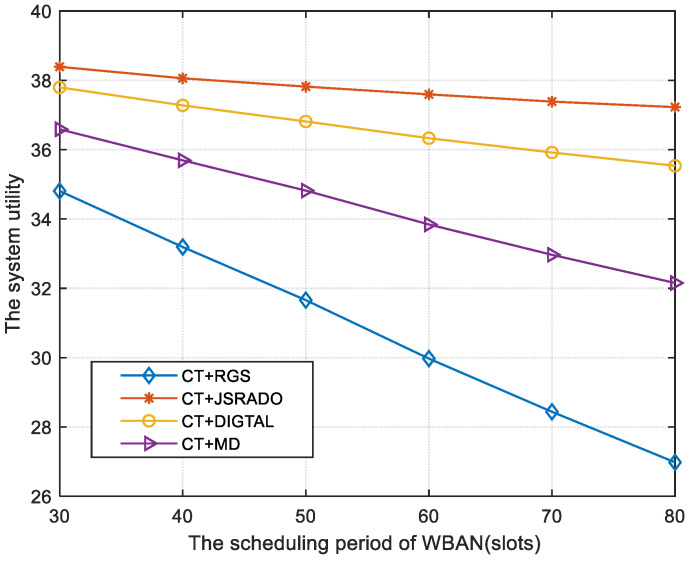
The system utility with different scheduling periods of the WBAN.

Figure 5 shows the tendency for the system utility to vary with the WBAN scheduling period. The system utility decreases with an increase in the WBAN scheduling period. If the number of time slots for the WBAN is increased, each patient will have more slots to update medical monitoring data packets, resulting in a huge size of the collected packets in the GUNs, which indicates a higher system cost as the transmission delay and the energy consumption of the GUNs become larger. For practical application, the length of the WBAN scheduling period should be adjusted according to the number of sensors, the size of packets, and the level of medical urgency of each sensor to enhance the system utility. As shown in Figure 5, the proposed strategy composed of the CT and the JSRADO algorithm can always obtain larger system utility compared with the RGS algorithm, the MD algorithm, and the DIGTAL algorithm.

**Figure 6:**
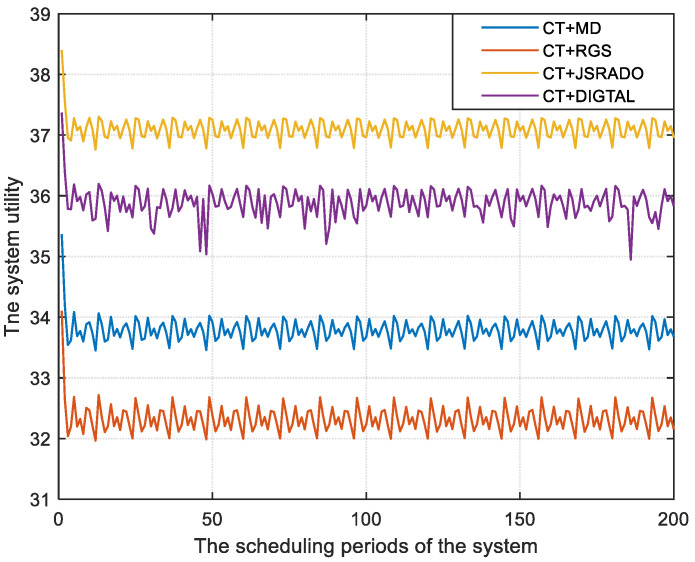
The system utility with different scheduling periods of the system.

The changes in system utility under different system scheduling periods are shown in Figure 6. It can be seen that the system utility is different in each scheduling period of the system. As the AoI of the monitoring data changes over time, the slot allocation for the WBAN and the data offloading for the ECN vary for different scheduling periods of the system. To maximize the system utility, the transmission opportunities in the WBAN scheduling period are always allocated to the sensors with a poor AoI, while the GUNs in the ECN always select the ESs with a lower cost to offload, which results in a slight difference in the system utility in each scheduling period of the system. However, Figure 6 shows that the strategy proposed in this paper always outperformed the other three algorithms over 200 simulated system scheduling periods.

**Figure 7:**
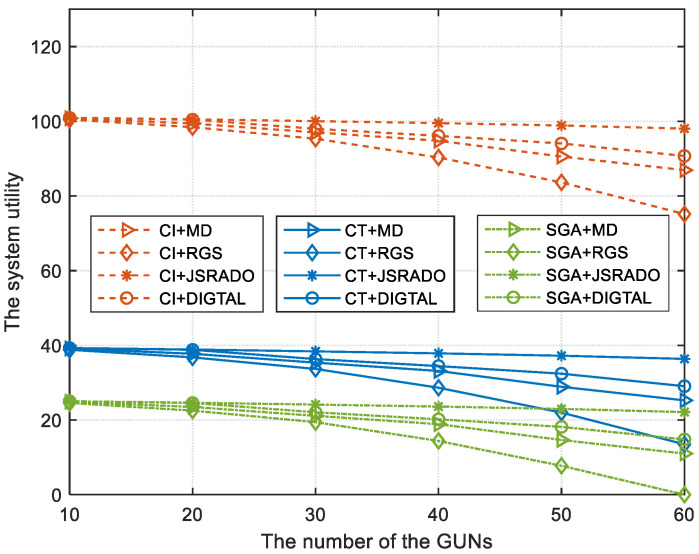
The system utility with different strategies.

Figure 2 and Figure 3 demonstrate the effectiveness of the proposed CT algorithm, while Figure 4, Figure 5 and Figure 6 demonstrate the superiority of the proposed JSRADO algorithm on the basis of the optimal contract. To verify the effectiveness of the strategy composed of the CT and JSRADO algorithm proposed in this paper, three incentive mechanisms and four data offloading algorithms were simulated as shown in Figure 7. From Figure 7, it can be seen that the combination of the CI and JSRADO algorithm can obtain the maximum system utility, while the combination of the SGA and the MD algorithm can obtain the minimum system utility. However, as the MC cannot be informed of all ESs’ participation willingness in advance in practical scenarios, the combination of the CT and the JSRADO algorithm may be suitable for resource allocation and data offloading in practical applications. Moreover, we find that the JSRADO algorithm based on the CT incentive mechanism can improve the system utility by almost two times compared with the data offloading JSRADO algorithm with the SGA incentive mechanism.

**Figure 8:**
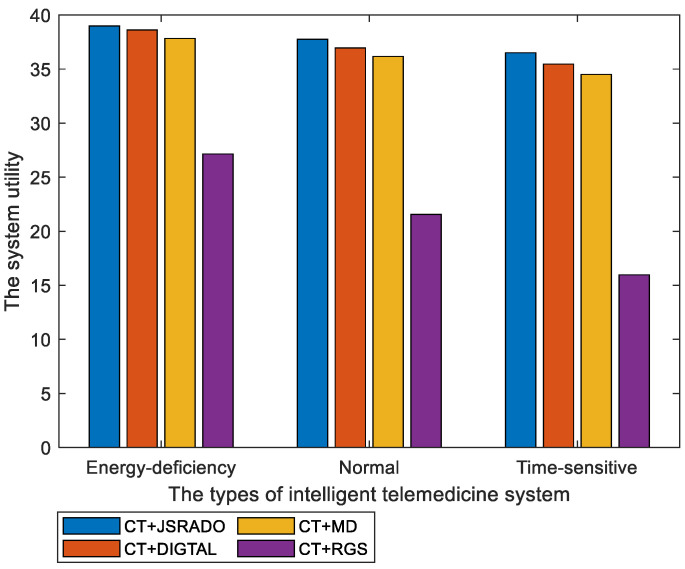
The system utility with different types of intelligent telemedicine systems.

Figure 8 illustrates the comparison of the system utility in different types of intelligent telemedicine systems. It can be seen that the system utility is different in the three types of intelligent telemedicine systems. In a normal system, the same weight is assigned to the two parameters, i.e., $\beta^{A}=\beta^{E}=0.5$. In energy-deficiency systems, energy consumption needs to be seriously considered to ensure sufficient energy supplies and to extend the working life of the system. Thus, the coefficients are set as $\beta^{A}=0.1$ and $\beta^{E}=0.9$. In time-sensitive systems, the medical information monitored by each sensor needs to be updated frequently. To improve the timeliness of the data updates, the parameters are set as $\beta^{A}=0.9$ and $\beta^{E}=0.1$. As shown in Figure 8, the strategy composed of the CT algorithm and the JSRADO algorithm always outperformed the other strategies in terms of the system utility.

## 6. Conclusions

In this paper, an edge-computing-assisted intelligent telemedicine system consisting of the WBAN and the ECN was considered. Based on the requirements of the patients and the utility of the MC, it was assumed that system utility is jointly determined by the MC’s utility and the system cost. Considering the selfishness of ESs, the CT algorithm was first utilized to encourage ESs to provide communication resources to maximize the utility of the MC. In addition, the JSRADO algorithm was proposed to minimize the system cost, where the cooperative game and the bilateral matching game were used to address the slot allocation problem for the WBAN and the data offloading problem for the ECN, respectively. Numerical simulations demonstrated the effectiveness of the strategy composed of the CT algorithm and the JSRADO algorithm in this paper with respect to the system utility.

## Figures and Tables

**Table 1:** Summary of key notations.

Notations	Description
N	The set of patients or WBANs or GUNs
M	The set of ESs
S	The set of sensors in each WBAN
K	The set of the medical urgency levels
u	Time slot allocation strategy
a	Collected packet offloading strategy
θ	Type of ESs
$\xi_{i}$	$\mathrm{Probability}\;\mathrm{of}\;\theta_{i}$
$q_{i}$	Communication resources contributed by ES i
$\pi_{i}$	Rewards for ES i
I	The total types of ESs
$G_{ij}(t)$	Generation timestamp of the data packet of the sensor j in WBAN i
$A_{ij}(t)$	The AOI of the sensor j in WBAN i at slot t
T	The scheduling period of the WBAN
$\delta_{k}$	The coefficient of medical urgency level k
$B_{i}$	The medical urgency of the packet collected by GUN i
$F_{s}$	Computing resources of the ESs
$u_{i,j}(t)$	Time slot allocation variable
$x_{ijk}$	Medical urgency class variable
$a_{im}$	Collected packets offloading variable

**Table 2:** Basic parameters of system simulation.

Parameters	Values
Computing resources of the ESs	30 GHz
Transmission power of the sensors	20 mW
Transmission power of the GUNs	0.5 W
Pass loss factor	3
Data size of medical monitoring packets	(100, 1000) KB
Computation intensity	(400, 800) CPU cycles/bit
Noise	−100 dBm
α,c,ε	$5*10^{6},1*10^{6},8$
θ	0.1, 0.2, 0.3, 0.4, 0.5 [22]

## Data Availability

Not applicable.

## Appendix A. Proof of Lemma 1

**Proof** **of** **Lemma** **1.** We first prove the “sufficiency”, i.e., when there is $\theta_{i}>\theta_{j}$, $\pi_{i}(\theta_{i})>\pi_{j}(\theta_{j})$ will be satisfied. Based on IC condition, there are:

(A1) $$\theta_{i}v(\pi_{i}(\theta_{i}))-cq_{i}(\theta_{i})\geq \theta_{i}v(\pi_{j}(\theta_{j}))-cq_{j}(\theta_{j})$$

(A2) $$\theta_{j}v(\pi_{j}(\theta_{j}))-cq_{j}(\theta_{j})\geq \theta_{j}v(\pi_{i}(\theta_{i}))-cq_{i}(\theta_{i})$$

Adding and simplifying Equations (A1) and (A2), then:

(A3) $$\left(\theta_{i}-\theta_{j})v(\pi_{i}(\theta_{i}))\geq (\theta_{i}-\theta_{j})v(\pi_{j}(\theta_{j})\right)$$

Since $\theta_{i}>\theta_{j}$, we have $v(\pi_{i}(\theta_{i}))>v(\pi_{j}(\theta_{j}))$. Based on the strict monotonicity of the v(π), $\pi_{i}(\theta_{i})>\pi_{j}(\theta_{j})$ is satisfied. Thus, “sufficiency” is proven. Then, we prove the “necessity”, i.e., when there is $\pi_{i}(\theta_{i})>\pi_{j}(\theta_{j})$, $\theta_{i}>\theta_{j}$ will be satisfied. Based on Equation (A3), we have:

(A4) $$\theta_{i}[v(\pi_{i}(\theta_{i}))-v(\pi_{j}(\theta_{j}))]\geq \theta_{j}[v(\pi_{i}(\theta_{i}))-v(\pi_{j}(\theta_{j}))]$$

According to the monotonicity of the evaluation function and $\pi_{i}(\theta_{i})>\pi_{j}(\theta_{j})$, we can find that $\theta_{i}>\theta_{j}$. Thus, “necessity” is proven. □

## Appendix B. Proof of Lemma 2

**Proof** **of** **Lemma** **2.** Proof the “sufficiency”. Based on Equation (A1), we have:

(A5) $$\theta_{i}[v(\pi_{i}(\theta_{i}))-v(\pi_{j}(\theta_{j}))]\geq c(q_{i}(\theta_{i})-q_{j}(\theta_{j}))$$

Since c>0 and $q_{i}(\theta_{i})-q_{j}(\theta_{j})>0$, we can get $\theta_{i}[v(\pi_{i}(\theta_{i}))-v(\pi_{j}(\theta_{j}))]>0$. According to the monotonicity of the v(π), $\pi_{i}(\theta_{i})>\pi_{j}(\theta_{j})$ is satisfied. Thus, “sufficiency” is proven. Then, we proved the “necessity”. Based on Equation (A2), we can get:

(A6) $$\theta_{j}[v(\pi_{j}(\theta_{j}))-v(\pi_{i}(\theta_{i}))]\geq c(q_{j}(\theta_{j})-q_{i}(\theta_{i}))$$

Since $\pi_{i}(\theta_{i})>\pi_{j}(\theta_{j})$ and the strict monotonicity of the evaluation function, $v(\pi_{j}(\theta_{j}))-v(\pi_{i}(\theta_{i}))<0$ is satisfied, i.e., $q_{i}(\theta_{i})>q_{j}(\theta_{j})$ is satisfied. Thus, “necessity” is proven. □

## Appendix C. Proof of Lemma 3

**Proof** **of** **;Lemma** **3.** By iterating the IC condition, we can obtain:

(A7) $$\theta_{i}v(\pi_{i}(\theta_{i}))-cq_{i}(\theta_{i})\geq \theta_{i}v(\pi_{1}(\theta_{1}))-cq_{1}(\theta_{1})\geq \theta_{1}v(\pi_{1}(\theta_{1}))-cq_{1}(\theta_{1})\geq 0$$

Equation (A7) shows that once the ES with participation willingness $\theta_{1}$ satisfies the IR condition, the other ESs automatically meet the IR condition. Thus, the IR constraint can be simplified as:

(A8) $$\theta_{1}v(\pi_{1}(\theta_{1}))-cq_{1}(\theta_{1})\geq 0$$

Thus, Lemma 3 is proven. □

## Appendix D. Proof of Lemma 4

**Proof** **of** **Lemma** **4.** Based on the IC condition, we can get:

(A9) $$\theta_{i}v(\pi_{i}(\theta_{i}))-cq_{i}(\theta_{i})\geq \theta_{i}v(\pi_{i-1}(\theta_{i-1}))-cq_{i-1}(\theta_{i-1})$$

(A10) $$\theta_{i-1}v(\pi_{i-1}(\theta_{i-1}))-cq_{i-1}(\theta_{i-1})\geq \theta_{i-1}v(\pi_{i-2}(\theta_{i-2}))-cq_{i-2}(\theta_{i-2})$$

Converting Equations (A9) and (A10) on the basis of $\theta_{i}>\theta_{i-1}$, we can get:

(A11) $$\theta_{i}v(\pi_{i}(\theta_{i}))-cq_{i}(\theta_{i})\geq \theta_{i}v(\pi_{i-1}(\theta_{i-1}))-cq_{i-1}(\theta_{i-1})\geq \theta_{i}v(\pi_{i-2}(\theta_{i-2}))-cq_{i-2}(\theta_{i-2})$$

Iterating Equation (A11), we have:

(A12) $$\theta_{i}v(\pi_{i}(\theta_{i}))-cq_{i}(\theta_{i})\geq \theta_{i}v(\pi_{i-1}(\theta_{i-1}))-cq_{i-1}(\theta_{i-1})\geq \ldots \geq \theta_{i}v(\pi_{1}(\theta_{1}))-cq_{1}(\theta_{1})$$

Thus, all DICs can be equivalently replaced by the LDICs as shown in Equation (32). Based on IC condition, there are:

(A13) $$\theta_{i}v(\pi_{i}(\theta_{i}))-cq_{i}(\theta_{i})\geq \theta_{i}v(\pi_{i+1}(\theta_{i+1}))-cq_{i+1}(\theta_{i+1})$$

(A14) $$\theta_{i+1}v(\pi_{i+1}(\theta_{i+1}))-cq_{i+1}(\theta_{i+1})\geq \theta_{i+1}v(\pi_{i+2}(\theta_{i+2}))-cq_{i+2}(\theta_{i+2})$$

Converting Equations (A13) and (A14) on the basis of $\theta_{i+1}>\theta_{i}$, we can get:

(A15) $$\theta_{i}v(\pi_{i}(\theta_{i}))-cq_{i}(\theta_{i})\geq \theta_{i}v(\pi_{i+1}(\theta_{i+1}))-cq_{i+1}(\theta_{i+1})\geq \theta_{i}v(\pi_{i+2}(\theta_{i+2}))-cq_{i+2}(\theta_{i+2})$$

Iterating Equation (A15), we have:

(A16) $$\theta_{i}v(\pi_{i}(\theta_{i}))-cq_{i}(\theta_{i})\geq \theta_{i}v(\pi_{i+1}(\theta_{i+1}))-cq_{i+1}(\theta_{i+1})\geq \ldots \geq \theta_{i}v(\pi_{I}(\theta_{I}))-cq_{I}(\theta_{I})$$

Thus, all UICs can be equivalently replaced by the LUICs as shown in Equation (33). □
